# Fast-tracking action on the Sustainable Development Goals by enhancing national institutional arrangements

**DOI:** 10.1371/journal.pone.0298855

**Published:** 2024-03-20

**Authors:** Mariam Akhtar-Schuster, Lindsay C. Stringer, Nichole Barger

**Affiliations:** 1 DLR Projektträger, Berlin, Germany; 2 Department of Environment and Geography, University of York, York, United Kingdom; 3 York Environmental Sustainability Institute, University of York, York, United Kingdom; 4 The Nature Conservancy, Arlington, Virginia, United States of America; 5 The University of Colorado, Boulder, Colorado, United States of America; University College Dublin, IRELAND

## Abstract

Six years remain to achieve the Sustainable Development Goals (SDGs). Despite some progress, institutional effectiveness for SDG achievement has not been delivered at a national level. Identification and establishment of an institutional framework to operationalise the 2030 Agenda within national plans, giving science-based coordination of SDG implementation a central role, is urgently required to accelerate progress. This paper tackles this challenge. Drawing on literature analysis, it asks: 1) What are the deficiencies in institutional national arrangements that hinder SDG implementation? 2) How can existing institutional deficiencies in SDG implementation be addressed? and 3) How can institutional changes support fast-tracking of SDG implementation processes at national level? Findings show that country-specific horizontal institutional arrangements are usually advanced. However, national visions to improve mainstreaming across decision-making at different levels to enable whole-of-government and whole-of-society approaches to SDG implementation are commonly under-developed. Deficiencies are due to poor systematic engagement of scientific and technical expertise in operational day-to-day communication, as well as in the design, validation, implementation, monitoring and reporting of domestic SDG-related multi-stakeholder actions. Vertical institutional arrangements are complex, and risk resource-consuming, uncoordinated implementation. Our analyses suggest countries may benefit from establishing a national, centralised independent scientific and technical coordinating body for SDG implementation at national level, within existing science-based institutional arrangements. Such a body would not be led by governmental processes but would provide technical support to government agencies. We argue that scientific and technical skills in data and information management and quality control are central to coordinated and evidence-informed support, and could help to accelerate national SDG implementation. Such a supporting body would also enable a more joined-up approach between stakeholders working in the areas of science and technology, government and practice, improving orchestrated science-based actions and their auditing across sectors and stakeholder communities at national and sub-national levels. It would further guide actions to reduce trade-offs within national sustainable development aspirations, and would facilitate consideration of diverse values in advancing towards a durable and just transformative future. Such efforts are vital given the rapidly closing window of time for SDG achievement.

## Introduction

The Sustainable Development Goals (SDGs) were adopted by United Nations members in 2015. This set of global commitments acknowledges the importance of integrating human and natural systems and moving beyond single-sector approaches in addressing sustainable development challenges. Such joined-up understanding has long been evolving in the environmental systems literature, which situates the environment at the nexus of the transformations necessary to support sustainability [1, 2]. Institutions influence and are influenced by policies; affect how decisions are taken; and shape dominant systems of governance. At the same time, institutions and associated governance systems are indirect drivers that can negatively and positively affect the management of land and the wider environment [3]. Thus, they play an important role in advancing progress towards development outcomes. National institutional arrangements are key in operationalizing the 2030 Agenda for Sustainable Development. In 2019, the Economic and Social Council of the United Nations (ECOSOC) acknowledged that governments are highlighting the SDGs in their national plans and policies, and establishing institutional arrangements to “help drive and also monitor progress towards the transformation needed in their economies and societies” [4]. The ECOSOC report nevertheless raised serious concerns that the necessary level of institutional effectiveness and resources, including a financial commitment to accelerate SDG implementation, has not yet been achieved at a national level. In autumn 2019, the political declaration of the high-level political forum on sustainable development under the auspices of the United Nations General Assembly stressed: “the urgent need to accelerate action on all levels and by all stakeholders, in order to fulfil the vison and Goals of the 2030 Agenda” [5]. The declaration mentioned ten areas where commitment is required, including the need for “[s]trengthening institutions for more integrated solutions” at all levels, but specifically at a domestic level [5]. The Ministerial Declaration of the high-level segment of the 2020 session of the ECOSOC and the 2020 high-level political forum on sustainable development again stressed slow progress in implementing the 2030 Agenda, emphasizing that accelerated actions should also include “efforts to build and strengthen more effective, accountable and transparent institutions” [6]. As of August 2022, 387 acceleration actions had been submitted by governments and organisations, seeking to accelerate action on one, several, or all SDGs, but without specifically addressing the need to strengthen domestic institutions in national SDG implementation [7].

The forms and functions of institutions to support sustainable development should be purpose-driven [8] and organized to meet specific needs, while their design should enable flexibility to adjust to emerging demands and changing circumstances, including changing environmental conditions. In this context, adjusting the organization and functions of national institutional set-ups is critical for SDG implementation, considering all sectors and stakeholders in an integrated whole-of-government and whole-of-society approach [9–11]. Learning from integrated analyses of land and environmental systems and the challenges and opportunities identified could help to operationalise these needs and is particularly timely. Later in 2024, the UN will convene the Summit of the Future on the theme, ‘Multilateral Solutions for a Better Tomorrow.’ The Summit’s aim is to reinforce the UN and global governance structures to better address old and new challenges and to formulate a Pact for the Future that would help advance the SDGs by 2030 [12]. The Summit of the Future offers a rare opportunity to bridge the considerable gap between the magnitude of the challenges the world is facing and national level actions. Insights we present here also inform ways forward to address those challenges, against the background that only six years remain to achieve the SDGs.

This paper addresses three major research questions that advance understanding of the institutional deficiencies and support fast-tracking of progress:

1. What are the deficiencies in national institutional arrangements that hinder SDG implementation that have emerged from scientific assessments in the land and wider environment sector?
2. How can existing national institutional deficiencies be addressed to support SDG implementation?
3. How can institutional changes support fast-tracking of SDG implementation processes at national level?

To address these questions, we draw on evidence from three Summary for Policymakers documents (SPMs) of key recent science-policy reports on environmental sustainability and land challenges [3, 13, 14], the Global Land Outlook (GLO) [15]; literature on existing institutional deficiencies and requirements for SDG implementation; and analyses and reviews of national institutional arrangements for implementing the 2030 Agenda for Sustainable Development from 93 countries [16, 17].

Our analysis first recognises that situations and thus, institutions, change continuously, a circumstance that should open plausible opportunities for integrating enablers in institutional build-up that provide continuity in implemented multi-stakeholder ‘national roadmaps’ [18]. Such continuity is important in monitoring actions to achieve progress, in maintaining outcomes of achievements towards domestic goals, and in ensuring accountability. Second, institutions should provide the flexibility to prepare for coordinated reengineering or reorientation of actions to optimize country-level SDG-implementation over time. Both aspects need to be driven by continuous tracking and evaluation of the impact of the measures taken, also to support their fast-tracking in practice [2, 8, 16] and cannot be addressed by structures within governments [18]. Instead, it requires adequate channelling of science-based expertise in the context of national SDG processes. As noted by the UN “[T]hese actors appear to be increasingly involved in promoting and monitoring the Goals, though there is scope for even greater engagement” [18]. Such science-based approaches would also build the basis for identifying adequate capacity-building in a national whole-of-society-driven SDG implementation process [18].

Policy integration, both horizontally and vertically, has long been indicated to support the kinds of collaborations and partnerships required to enhance implementation of complex policy issues such as environmental challenges and the SDGs [19–21]. [22] considers integration as simply “the management of cross-cutting issues in policymaking that transcend the boundaries of established policy fields”. Horizontally, this means taking a more joined-up approach within a governance level and bringing together different sectors, while vertical integration relates to interlinkages across different governance levels. Authors have described policy integration variously as a normative principle, a process, an outcome, an output or as an organisational and structural concern [23–25]. Although the literature often presents integration as a necessity (and implicitly through emphasising coherence, it has been adopted in SDG target 17.14: ‘enhance policy coherence for sustainable development’), it can also bring challenges, helping to explain why many countries continue to grapple with this issue in practice. Relatively little remains known about the operationalisation of integration, and the conditions under which certain actions deliver the desired goals [25]. Challenges include blurred lines of accountability, difficulties in assessing and measuring impact and effectiveness, increased opportunity costs associated with management and staff time, as well as the costs incurred with cross-cutting approaches and structures as part of the transition toward integration [26]. The extent to which these critiques and challenges apply depends on the pre-existing structures and systems, the incorporation of additional policy targets, and the need to integrate governance and institutional arrangements [27]. In this sense, one approach to such integration is a whole-of-government approach, while Finland points toward phenomenon-based budgeting and resource allocation, and systematic integration of SDGs with “the Government Programme, legislation, budgeting, performance management and human resources management” [28]. The country also seeks to integrate sustainable development with regional processes in developing a new regional administration.

## Materials and methods

This research took a desk-based approach, grounded in the identification and analysis of published scientific peer reviewed literature, a sample of three science-policy reports that place environmental and land concerns at their core, and review of other United Nations documents.

To address the first research question, we particularly focused on the evidence in scientific reports developed for science-policy interfaces with a land component. Land is increasingly recognized as a connector between environmental issues and nature-based solutions are integral to the overall design of policies, measures and actions for sustainability [29]. Recent scientific assessment reports and associated summaries for policymakers (SPMs) developed by international science-policy interfaces in environmental issue areas that cross-cut land have directly informed governments about important institutional deficiencies and requirements for SDG implementation at country level. As such, it provided a useful boundary for the body of work for us to investigate, while the SPMs of the IPBES and IPCC reports have been approved by governments on a line-by-line basis, so their findings already have a policy-strategic tinge. Our sample includes the Global Land Outlook [15] by the UNCCD, which supports efforts to operationalise SDG target 15.3 on Land Degradation Neutrality (LDN) [30, 31]; the Intergovernmental Science-Policy Platform on Biodiversity and Ecosystem Services (IPBES) reports that assess Land Degradation and Restoration [32] and global biodiversity and ecosystem services [33], and the Intergovernmental Panel on Climate Change (IPCC) Special Report on the links between Climate Change and Land [13]. Each of the sampled assessments also directly consider governance and institutional deficiencies that need to be addressed to progress towards sustainable development. Together, these reports and their converging recommendations offer useful lessons in further shaping institutional arrangements for SDG implementation at country level.

The three SPMs’ challenges [3, 13, 14] were reviewed first, manually through reading and review, and using keyword searches within the documents. The process was complemented by a similar screening of the GLO [15]. [34] developed a “four-dimensional typology for the categorization of national bodies for SDG-implementation”, which considered: (i) political leadership, (ii) horizontal integration, (iii) vertical integration, and (iv) societal integration. We categorised the institutional deficiencies into three groups that broadly mapped onto these but which better accommodated aspects such as inclusive approaches that are central to land systems approaches. Our categories therefore considered: (i) fragmented policy and policy leadership across sectors (horizontal institutional arrangements), (ii) missing or disconnected institutions and other resources at different national scales (vertical institutional arrangements), and (iii) lack of or missing institutional anchors for inclusive participatory approaches. Findings on institutional deficiencies mentioned in the science-policy reports were supplemented with searches for recent publications using Google and Google Scholar.

We used the search terms “institutional arrangements” AND/OR “implementation” AND/OR “SDG” AND/OR “projects” AND/OR “United Nations”. The same scientific assessments and search engines were then used to identify key enablers and evidence of work undertaken on accelerating or fast-tracking SDG implementation, using the terms “definition” AND/OR “fast-tracking” AND/OR “accelerating” AND/OR “SDG” AND/OR “project*” AND/OR “United Nations” in different combinations, to address our second research question. We incorporated the IEEEXplore Digital Library to extend our search of the meaning and use of terms such as “accelerator” and “fast-tracking”, because no specific definitions could be found for these in the other documents we had identified. We conceptualise “fast tracking” as a sequence of parallel, integrated and continuously coordinated and reengineered processes that compress the time that would otherwise be needed in a series of successive steps (see [35]). Searches continued with Google and Google Scholar to also identify, review and analyse recent peer-reviewed articles and UN documents on institutional arrangements for SDG implementation. Priority was given to material published after adoption of the SDGs in 2015.

To address the second and third research questions, we used the three groups of institutional deficiencies and requirements, and the set of key enablers to support fast tracking identified from the literature, to analyse available case studies on progressing institutional arrangements for SDG implementation at the country level. Ninety-three compendiums of national institutional arrangements for implementing the 2030 Agenda for sustainable development were assessed, drawing on two major reports: [16, 17]. These reports provided snapshots of evolving national institutional set-ups. While available data and information on national progress was rather generic and incomplete, the simple and standardized template of country compendiums allowed comparisons in terms of: (i) national strategies and plans; (ii) institutional arrangements; (iii) local authorities; (iv) parliament; (v) engaging and equipping public servants; (vi) civil society and the private sector; (vii) monitoring and review; (viii) engaging supreme audit institutions; and (ix) budgeting [16, 17]. Findings were arranged against our three categories and the enablers emerging from the literature. We then bundled the enablers in such a way that they could support institutional arrangements at country-level to coordinate a dense set of activities in condensed timeframes to improve the speed of SDG implementation and thus support fast-tracking.

## Results

### What are the deficiencies in national institutional arrangements that hinder SDG implementation that have emerged from assessments in the land and environmental sector?

Analyses of the horizontal institutional deficiencies according to the four science-policy assessments highlighted governance fragmentation as a key challenge, leading to siloed responses: “Most policies directed at addressing land degradation are fragmented and target specific, visible drivers of degradation within specific sectors of the economy, in isolation from other drivers” [14]. Such fragmentation means an overarching national narrative for implementing domestic environmental sustainability goals is lacking, while narrow, poorly coordinated responses focus on the short term. These aspects set the scene for silos and competing national targets [3, 13–15]. Furthermore, policy institutions and governance systems were found to focus on reactive responses [14, 15] failing to address the causes of environmental issues in a proactive and resource-efficient way [14, 15].

Vertical domestic institutional deficiencies to effectively address SDG-relevant environmental issues were also noted. Institutions at national, sub-national and local levels were found to be insufficiently resourced/financed and/or to lack expertise and competencies including skills, knowledge and technology [3, 14], while important institutions and stakeholders were missing or disconnected [3, 14, 15]. For example, land tenure systems (registry and cadastre) need to be included to provide greater planning security to land users, as “sustainable use is heavily influenced by the security of people’s rights to land resources” [15]. [14] emphasizes the need to improve “interactions amongst policies and land and resource-management practices to address different Sustainable Development Goals and other multilateral agreements, and the consequences of these efforts for land degradation and restoration outcomes”, and create enabling conditions to avoid, reduce and reverse land degradation “regarding technical capacities, technologies, data and information access, knowledge sharing, decision support tools and institutional competencies”. The IPBES report [3] highlights the need for better consideration of the participation of Indigenous peoples and local communities and their knowledge in national measures for conservation and sustainable use. The IPCC report [13] underscores that “… efforts can be more effective when policies support local management of natural resources, while strengthening cooperation between actors and institutions, including at the international level”, and notes “… a lack of engagement between stakeholders at different scales”. [13] also highlights that “[C]oordination with other sectors, such as public health, transportation, environment, water, energy and infrastructure, can increase co-benefits, such as risk reduction and improved health”.

These challenges hamper vertical information flows, exposing a gap in terms of coherent and interconnected policies (top-down), and a lack of consideration of multi-stakeholder needs in the design, implementation and monitoring of SDG measures (bottom-up) [3, 14]. Furthermore, reports showed a lack of coordination of domestic institutional processes which include multiple stakeholders (such as line ministries, local authorities, civil society and private sectors, academia, national agencies and institutions of higher educations, Indigenous groups and local communities and other marginalized or vulnerable groups, alongside international organisations such as UN bodies and development agencies [3, 13–17], risking duplication of efforts, or worse, undermining of one another. Major expertise required from these groups (such as scientific, technical and business data, as well as Indigenous and local knowledge), is then missed, illuminating the need for improvements to participatory approaches. Governance systems that fail to support participatory approaches can exacerbate social exclusion, social injustice and marginalise key stakeholders—those whose knowledges and perspectives are needed for designing and implementing SDG measures: “Coordinated action to implement the response options will be required across a range of actors, including business, consumers, land managers, Indigenous peoples and local communities and policymakers to create enabling conditions” [36]. Exclusion can enhance risks of trade-offs between diverse national interests as well as consumer needs and associated production chains and networks, reducing the understanding and acceptance of required measures and associated resources at different national levels. This in turn can lead to conflicts and social injustice in the design and implementation of measures [14]. Overcoming these barriers requires “[I]institutional coordination, multi-stakeholder engagement and the development of governance structures that bridge different government functions, types of knowledge, sectors and stakeholder groups” [37]. All the science-policy reports we analysed reiterated this latter point multiple times (see S1 Table).

### How can existing institutional deficiencies be addressed?

#### Horizontal institutional improvements

Arrangements that ensure engagement and collaboration between multiple stakeholders strengthen cooperation between actors and institutions [3, 13–15]. Such actions can also reduce the negative social impacts that can emerge from conflicting demands and values in different sectors: “The effectiveness of decision-making and governance is enhanced by the involvement of local stakeholders (particularly those most vulnerable to climate change including Indigenous peoples and local communities, women, and the poor and marginalised)” [13]. UNSDG [38] shows in its interim report that this approach is paramount to leaving “no one behind”. Indeed, multi-stakeholder involvement is a “prerequisite for reducing trade-offs, enhancing alignment and harnessing synergies among decision-making areas” [14]. Institutions and governance systems engaging with stakeholders can benefit from measures that address land tenure, gender equality and investment in infrastructure, through timely consideration of a more complete range of societal opinions and needs in planning and associated implementation processes, thus enhancing the quality of both horizontal and vertical institutional arrangements. The IPBES [3] further suggests that community-based conservation institutions and local governance (often informal), can, at times, be more effective than formally established arrangements.

Analyses of the science-policy assessments further show that strong national vision and leadership, as well as strong coordination among national institutions and participation, are necessary for effective, inclusive country level SDG implementation. IPBES points out that: “[L]and degradation is rarely, if ever, the result of a single cause” and the same applies to other sustainability challenges. Such ‘wicked’ problems can “only be addressed through the simultaneous and coordinated use of diverse policy instruments and responses at the institutional, governance, community and individual levels” [14]. A mix of policies and instruments that are horizontally harmonized and mainstreamed can provide a national agenda for sustainable development across sectors and ministries, helping to ensure that trade-offs in actions between different national efforts for nature conservation, land rehabilitation/ restoration, agricultural production/ climate change mitigation and adaptation are avoided, or at least reduced and moderated [13, 14]. Coherence and harmonisation across and between policy mandates that consider a common national understanding and shared responsibility across institutions and sectors, is prerequisite for urgent, inclusive and integrated sustainable development and management approaches: “Strengthened multi-level, hybrid and cross-sectoral governance, as well as policies developed and adopted in an iterative, coherent, adaptive and flexible manner can maximise co-benefits and minimise trade-offs, given that land management decisions are made from farm level to national scales” [13]. Such efforts warrant continuous efforts and resourcing over the long term, including the development of institutional capacities, securing of finance, and incorporation of “knowledge from various systems, including the sciences and sustainable Indigenous and local practices” [3].

Our analyses of all 93 country compendiums [16, 17] on existing national strategies used or planned to align existing policies and plans with the SDGs, or to develop new ones in light of specific national circumstances, showed varying levels of progress or are not yet in place. This was also confirmed by [18] which sampled 24 countries. However, the efforts made by most governments to provide some strategic framework for the SDGs to support their domestic implementation show that Agenda 2030 and the SDGs need to be nationalized to some degree, alongside associated provision of political guidance and oversight. At the time of analysis, 27% of the 93 countries stated that the SDGs (to some degree) are aligned with existing national development principles; 43% have integrated country-relevant SDGs into national policies and plans; 3% are in the process of (further) integrating the SDGs into existing national policies and plans; and 10% will use Agenda 2030 to develop future national policies and plans. Several countries show a combination of the above processes.

We further analysed the 93 country reports against engagement of different ministries, assessing the extent of a cross-sectoral, whole-of-government approach in SDG implementation. Available data suggests 82% of governments have identified line ministries responsible for implementation of one or more SDGs, although different ministries are engaged in SDG processes to different degrees, and some countries (6%) indicate that cross-sectoral SDG responsibilities are planned. However, for several countries, the information provided was insufficiently specific to comprehensively assess the extent of mainstreaming and SDG nationalisation. Our analyses showed that 88% of the countries that submitted reports have already adapted or are planning a whole-of-government approach by involving different ministries in national SDG processes: a crucial first step to ensure common national acceptance across sectors and provide political guidance and oversight through horizontal coherence in policy decisions. It is also important to acknowledge what is missing from the data set, however. Those countries that did not submit voluntary national reviews might lack capacity and be less well positioned to implement the SDGs and mainstream them into national processes. This highlights an issue for further investigation.

Even if there is a strong national commitment towards Agenda 2030, national political guidance and oversight may be weak, particularly early in the nationalisation process. Countries may consider only specific aspects of the Agenda 2030 which already align with their existing policies and plans, without giving priority to analysing the appropriateness of existing national polices and plans for attaining sustainability. This was the case for countries including Albania, Benin [17], Cambodia, Cameroon, Côte d’Ivoire, Guatemala, Iceland, Palau, and Republic of Congo [16]. Assigning a specific SDG to a specific line ministry based on its respective area of competence can nevertheless exacerbate silo effects and trade-offs when implementing one or more SDGs. Another issue that can hamper the speed of SDG implementation (e.g. as found in Bhutan [17], Colombia [17] and Republic of the Congo [16]) lies in the limited temporal scope of the primary national framework used for integrating the SDGs in domestic sustainable development policies, some of which end ahead of 2030. Some countries (e.g. Bhutan, Guinea, Latvia, Mali and Senegal [17], and Ghana, Oman and Timor Leste [16] are implementing the SDGs through a decentralized sub-national approach that can empower authorities and promote the role of local communities in implementing national sustainable development objectives. While this has its advantages, it may detach SDG processes from government processes and could hinder the up- and out-scaling of effective measures.

The World Public Sector Report 2021 [18] clusters capacity building requirements for policy integration needs in the following three dimensions of horizontal integration: *“the promotion of collaboration*, *coordination and new ways of working together across organizational boundaries”*. Our findings suggest that coordinated steps to develop, implement and monitor country-specific actions will be required.

Another key aspect to ensure national guidance and oversight in the implementation of the 2030 Agenda is by informing policy processes through science-policy interfaces [18]. This could be achieved by identifying a national, centralised, independent scientific and technical coordinating body for the day-to-day work in domestic SDG-implementation, and may be more flexible to adjust to changing needs if it were established within existing science-based arrangements outside government structures. Such a body could help ensure coherence in national SDG implementation across scales and enable participatory planning and monitoring. It could also incentivse data generation and data quality control for reporting, if necessary, reengineering SDG implementation measures on the basis of best available evidence from different disciplines and knowledge domains. This would support continuous identification of data and knowledge gaps, as well as gaps in other necessary resources, including financial resources, training needs and development of necessary skills–core aspects highlighted as important within the scientific assessment reports. At present, such a body is largely absent from the 93 country compendiums, with governmental lead entities assigned coordination roles and only meeting occasionally. Indeed, a 2019 paper of the Think Tank of the European Parliament (which helps shape EU legislation) shows only a few countries have an institutionalised interface mechanism between science and policy for the SDGs, pointing to planned or existing national initiatives for independent mechanisms [39]. Germany provides an example of the development of an independent council for sustainable development that works with experts from different areas of society to support SDG implementation, while the French Ministry of Research has set up a working group to look at the role of science in implementing the SDGs, indicating that independent coordination and review arrangements that can inform government processes are anticipated to deliver benefits.

#### Vertical institutional improvements

[13] states that the “Appropriate design of policies, institutions and governance systems at all scales can contribute to land-related adaptation and mitigation while facilitating the pursuit of climate-adaptive development pathways”. Opportunities for improving vertical institutional arrangements by ensuring that they are interconnected, resourced, and adaptive to the necessary requirements for SDG implementation (which can change over time), are supported by discussions in the literature about the need for flexible institutional arrangements within a polycentric governance system [34, 40]. Development of institutional and individual capacities to design, implement and monitor SDG progress, as well as ensure the suitability of implementation instruments over time and their integration into ongoing institutional evolution, is a continuous process; one that will have durable impacts on national investments, particularly national budgets (see [10]). In the context of limited resources, particularly given the economic impacts of the COVID-19 pandemic and other current economic pressures, efficiencies will be needed to prioritize actions to achieve the SDGs, including within a particular SDG or SDG target, potentially considering triage approaches [41]. This will require scientific evidence, coordination and monitoring to ensure that trade-offs do not emerge or deepen between prioritized SDGs and less prioritised goals and targets. Institutional capacities will also have to be enhanced in many countries to help them to consider SDGs in budgetary debates. [42] point toward how this could be done.

**Fig 1:**
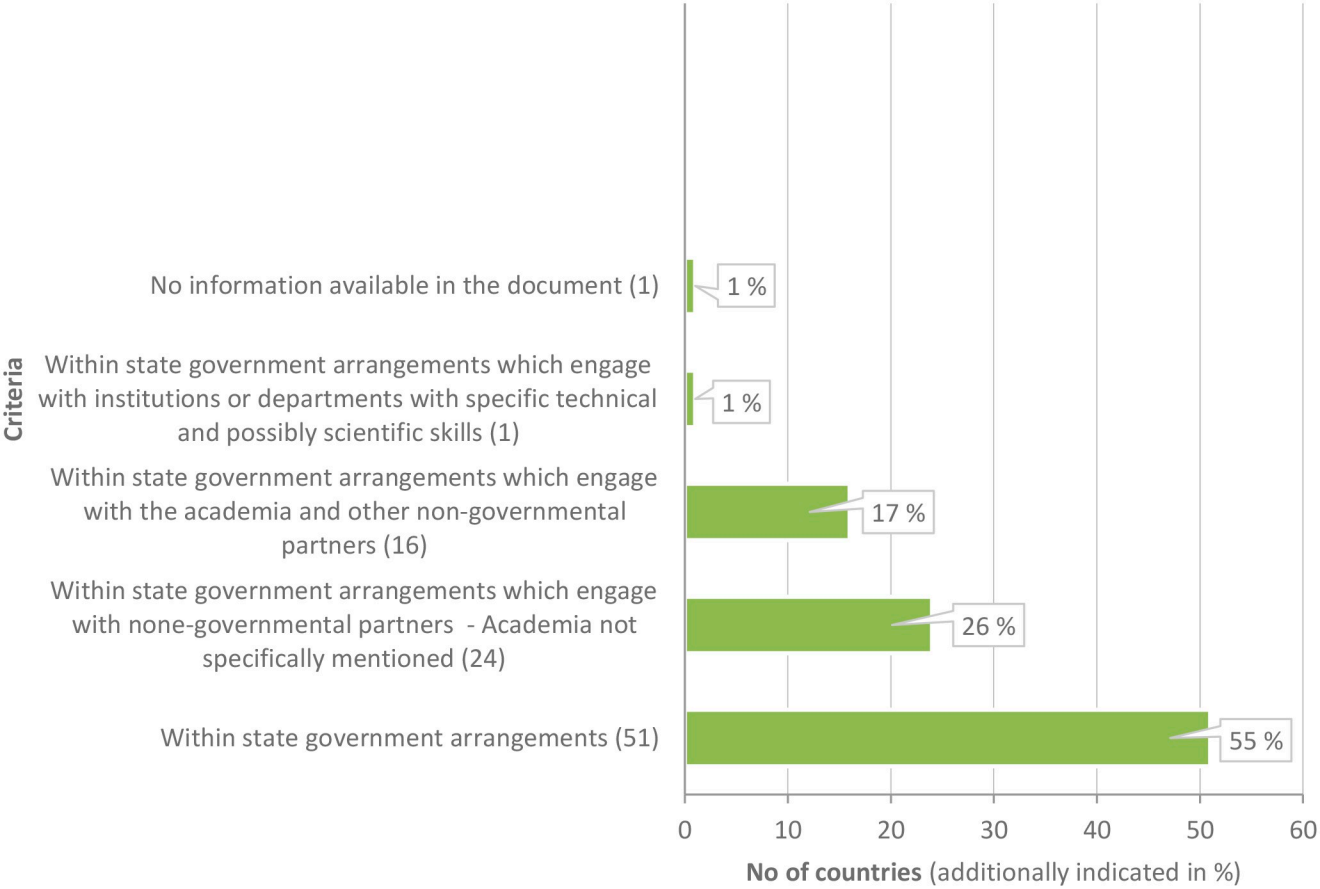
Location of the national lead and coordination entity for SDG implementation (existing /planned). Information sources: [16, 17].

Vertical institutional arrangements for SDG implementation in the 93 national compendiums were diverse and complex. Information on interconnectedness in work and communications between institutions was rudimentary and thus unclear [16, 17] In countries that provided more in-depth information, a national “lead and coordination point” has been or will be established in governmental arrangements, commonly overseen by a leading policy body (e.g. the Prime Minister’s office, Cabinet office, or government councils (Fig 1).

S2 Table shows that technical expertise is provided to government processes in various forms, including via technical committees, which are partly established within relevant ministries, and can include representatives of ministries, and /or national institutes and experts from the scientific and technical arena (academia). While co-design, co-implementation and co-monitoring of national SDG implementation measures (key collaborative aspects of governance, vital for success) emerged from all the analysed science-policy reports [3, 14, 15], it remains unclear how a policy-informing science-based coordination body can connect with complex national institutional set-ups, and with the diverse national stakeholder community on an operational basis to support such collaborative efforts. Given that most institutional arrangements are new, learning by doing (albeit time consuming) may be one option for their evolution toward improved effectiveness.

**Fig 2:**
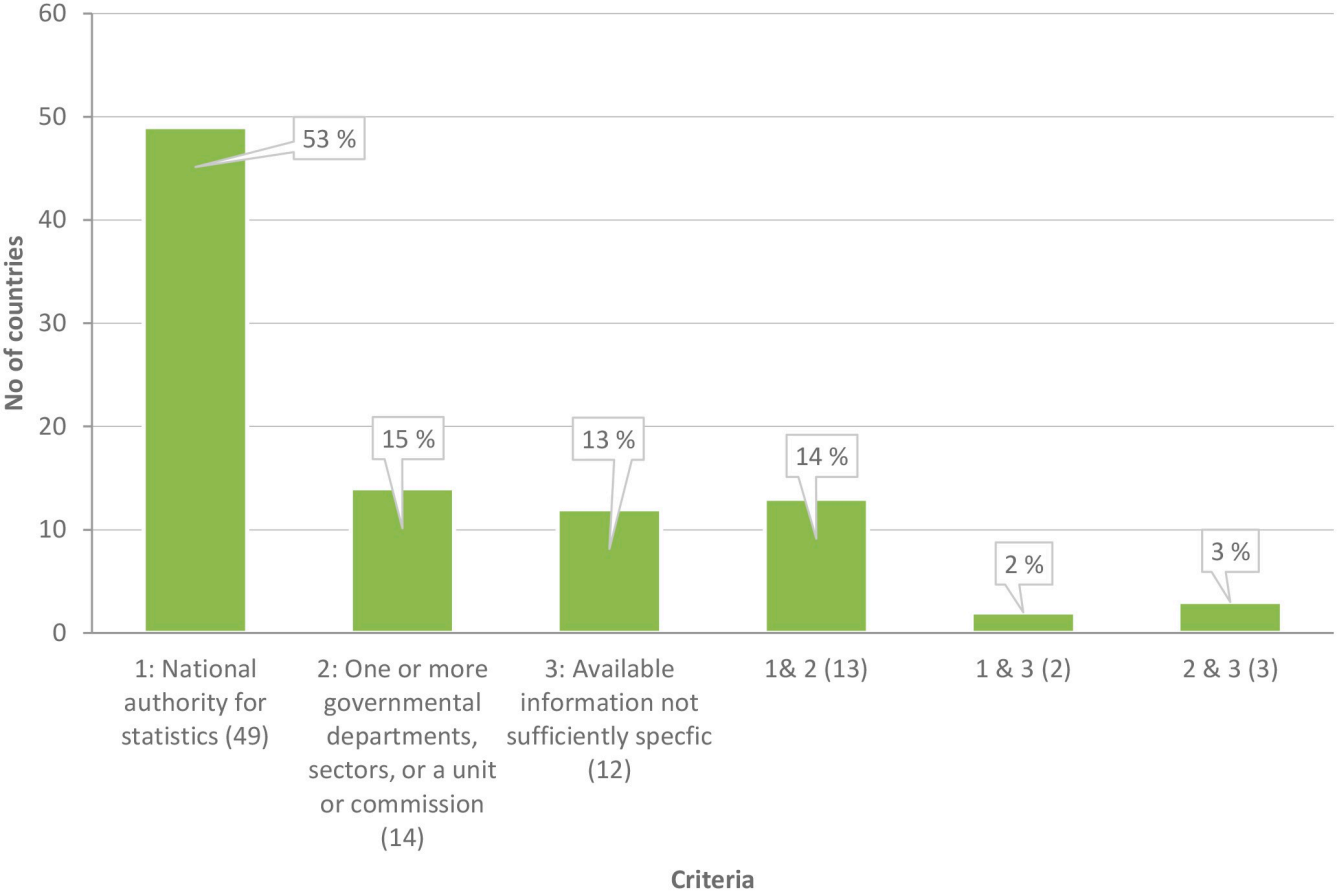
Responsibility for coordinating national monitoring and review, including identification of data and indicators pertaining to the SDGs (planned / existing). Information sources: [16, 17].

[3, 14] and [15] also emphasize the need to improve the competencies of national level institutions to gather and synthesize data, monitor knowledge and the effects of implemented measures, and to support efforts to up-scale local successes into large-scale transformative initiatives. Analyses of the 93 compendiums show that in the majority of country cases, where the information provided was sufficiently specific, the primary responsibility for reviewing and monitoring SDG implementation, and identifying indicators pertaining to the SDGs, lies or will lie with national statistical agencies, sometimes in collaboration with other governmental offices (Fig 2). National statistical agencies are vital actors for administering basic national SDG-related data and verifying the quality of existing data and information, but accessibility of this data needs improvement and is only starting to be addressed through initiatives such as the Federated Information System for the SDGs (FIS4SDGs) developed by the United Nations in partnership with ESRI. Using data and information to co-design, co-implement and co-monitor SDG measures within the principles of whole-of-government and whole-of-society approaches, requires coordination and operationalisation of actions via an institutional system, particularly also to capture insights from different forms of knowledge and in order to model and project future scenarios. Such a system should also be capable of coordinating data and information flows, identifying multiple knowledge gaps and knowledge requirements for implementation purposes, and if required, fast-tracking measures by institutions that operate at different national levels. It should also enable where agencies are duplicating data collection. Some countries have realised the need to give science more weight in domestic SDG processes [16, 17]. Besides engaging national authorities for statistics in SDG-related data, indicators, monitoring and auditing activities, Armenia [17]; Azerbaijan [16]; Colombia [17]; Hungary [17]; South Africa [16] and Turkmenistan [16] have also established national structures that give science and science-based approaches that integrate diverse knowledges a more central role.

The importance of stakeholder inclusion in achieving a whole-of-government and whole-of-society approach for SDG implementation is reflected in all 93 country compendiums. All countries acknowledged the role of civil society and the private sector for SDG implementation. Involvement of civil society groups was either planned or existing in the form of a loose collaboration via national awareness-raising days, media outreach, web portals, informational workshops media outreach, or through more in-depth collaboration in governmental processes, e.g. as participants of national consultation meetings, stakeholder fora, technical groups, or members of national committees.

Government collaboration with the private sector is just as diverse in the different countries [16, 17], and strategically led by ways to mobilize and promote domestic financing of SDG actions by incentivizing the private sector to participate in SDG implementation. Several countries emphasise public private partnerships as a way forward to mobilize resources (e.g. Armenia, [17]; Bahrain, [17]; Benin [17]; Bosnia and Herzegovina [16]; Burkina Faso [16]; Cabo Verde [17]; Ghana [16]; Jamaica [17]; and Slovakia [17]; while regional and international funding (including that from regional development banks and the World Bank) will also be important to secure national level implementation activities. Despite this, the lacking, or generic, descriptions of the involved institutions and stakeholders, do not allow assessment of the potential efficacy of the national participatory approaches; an aspect seen by all science-policy reports as fundamental for successful SDG-implementation [3, 13–15], particularly because these national arrangements are fairly new. Realizing their importance for vertical domestic implementation processes, existing loose arrangements can provide space and flexibility to further develop collaborations according to specific national institutional and governance needs. However, learning from ongoing national processes that have already laid the foundations for strong integration of civil society in concrete national planning processes is necessary. In Australia for example, civil society together with the Department of Foreign Affairs and Trade is engaged in internal SDG planning processes and initiatives [17]. Guinea invites representatives of civil society and the private sector to contribute to sector-specific policies [17], while Jamaica involves representatives of civil society and the private sector in SDG-related decision-making processes [17]. These experiences point toward a need to establish a national, centralised independent scientific and technical coordinating body for SDG implementation at national level, within existing science-based institutional arrangements that is authorised by, yet independent from government, to coordinate stakeholder consultations and ensure appropriate involvement in government-led processes from decision-making through to implementation at different governance levels.

### How can institutional changes fast-track SDG implementation processes at national level?

Addressing the previous questions has provided insights into current institutional deficiencies and how they can be improved. This section examines how national level institutional changes not only advance SDG implementation, but can accelerate and fast-track it. We point to key institutional elements (‘enablers’) that can strengthen domestic coordination of institutional and multi-stakeholder engagement. These efforts can streamline national SDG-implementation and support fast-tracking.

We suggest that fast-tracking measures essentially include capacity building in SDG learning, research and knowledge, and planning, but are by no means limited to such actions. The World Public Sector Protocol 2021 provides clear examples on such capacity building aspects already in place in different countries, which could be collated and analysed to inspire other countries [18]. Such capacity building measures must, however, be part of a coordinated, broader approach that includes day-to-day implementation, and engineering and monitoring measures to ensure that the actions taken are complementary, mutually reinforcing, generate synergies and are accountable.

In September 2019, the German Council for Sustainable Development (RNE), together with the Finnish Sustainability Commission and in cooperation with the UNDESA and the Stakeholder Forum, founded the Global Forum for National SDG Advisory Bodies to advise governments on effectively accelerating national and local solutions and approaches to sustainability [43]. The literature offers important lessons, suggesting that a national, centralised, independent scientific and technical coordination body may be needed to: coordinate communication among and engagement with diverse national actors; monitor the consideration of national policy visions, decisions and roadmaps throughout SDG implementation processes; secure scientific creditability of actions; ensure the availability and management of data, information and knowledge and direct actions across national scales; and provide an institutional set-up that can fast-track the co-design, co-implementation and co-monitoring of nationally prioritized SDG-related measures [9, 10, 44]. Similar processes have been followed in the environmental and land sectors.

Although several country compendiums do not explicitly mention this kind of independent coordination unit covering the enablers, their national institutional efforts for SDG implementation show the need for such a body. The majority of coordinating activities are currently placed under a political national entity, whereby academia and science are involved to varying degrees. S3 Table summarizes those national institutional efforts that give science more responsibilities in domestic SDG processes.

Some countries have nevertheless taken a strong science- and technology-based approach, even without such a centralised coordinating system. However, the operationalization of national and local measures to fast-track action on the ground will require central national coordination through such a body as those seen in Turkmenistan and Armenia, to effectively bundle measures, saving time by setting up parallel processes. These may include the series of complementary parallel actions which consider the minimum set of criteria used by UNDESA to select SDG Acceleration Actions (S4 Table), and can be either continuous throughout the fast-tracking process, or time limited. If countries are able to establish a national, centralised, independent scientific and technical body to coordinate SDG implementation, it may help them to optimize their vertical institutional arrangements and to support fast-tracking of national actions to meet the 2030 Agenda for Sustainable Development, particularly if they are bundled (Table 1).

**Table 1:** Bundles of measures to fast-track SDG implementation. UNDESA criteria are in bold, italic text.

Co-design bundle	Co-implementation bundle	Outputs, outcomes and further actions
Develop a strategic tool to support identification of priority actions required within the national vision for sustainable development show ***reflect interlinkages among goals and contribute to policy coherence***.	Enable and stimulate top-down and bottom-up debates to overcome sector silos, to identify ***reasonable means of implementation such as finance*, *technology or capacity building*** to warrant longevity and sustainability of the initiative, thereby also ensuring coordinated multi-stakeholder communication and engagement in co-implementation, and associated co-monitoring and if required co-reengineering of actions.	Analyse the efficacy of national actions at all geographic levels in meeting the SDGs.
Identify science, and other expertise as well as technology, data/information and partners, including government, civil society, private sector etc…, to ensure a country-as-a-whole approach in the co-design.	Develop capacities at all levels to ensure ***longevity and sustainability of the initiative***, and the scaling up best practices, as well as the recording of lessons learned.	Provide science-based evidence and actionable recommendations to the political agenda to scale up processes, including science-based evidence on how the fast-tracked initiatives have or can support ***interlinkages among goals*** and targets and **contribute to *policy coherence*** in addressing trade-off aspects and enhancing synergies.
Tailor specific fast-track SDG actions embedded in national or local circumstances which consider the ***SMART Criteria (specific*, *measurable*, *achievable resource based*, *and time-based deliverables)***.	Report on progress to policy and other decision-makers, funders, and the public through the use of different reporting formats. This will include the identification of quality indicators for regular monitoring, and analysing achievements against the national aspirations for sustainable development	Provide science-based evidence on how national initiatives support the implementation of the UNCCD, CBD and UNFCCC.
Analyse demands to support processes which decouple economy from unsustainable practices, and address non-market needs.	Ensure resource oversight and monitor use	
Analyse the availability and quality of the required data and information, and outline pathways to handle poor or absent data and knowledge.	Report to the government, local governments and the parliament, were national decisions or laws deviate from national sustainability principles, and the ***principles of the United Nations Charter and the 2030 Agenda***.	
Identify and ***build on existing successful efforts/initiatives (scaling up*, *new phase*, *etc*.*)***, and institutional arrangements (formal and informal), and network them and stakeholders to ensure that no duplication of tasks, mandates or actions use up limited resources for fast-tracking.		
Identify and attract domestic funding and international resources (financial) for fast-tracking SDG-implementation to ensure durability of actions, outputs and outcomes, e.g. through the development of an SDG narrative.		
***Provide information on the actions*** (e.g. ***website*, *contacts***).		

Sources: [3, 9, 10, 13–16, 34, 42, 45–48]

The science-policy reports we analysed showed that national institutional arrangements and multi-stakeholder engagement are fundamental for meeting the SDGs. Against the vertical and horizontal institutional requirements outlined in these reports, analysis of the 93 compendiums revealed that most reporting countries’ (horizontal) institutional arrangement show reasonable progress in mainstreaming a national sustainable development agenda. This should help to build cross-sectoral coherence for the development of national policies for fast-tracking actions for SDG implementation.

However, assessing the quality of the connectivity of existing or planned institutions, human and other resources that are involved at different levels (vertical institutional arrangements) is difficult because of limited information and due to their complexity. Also, some institutions are still in the planning phase. Nevertheless, most countries are explicitly striving for a whole-of-country approach, by engaging or planning to engage with local authorities, the civil society and the private sector, parliament and public servants, monitoring and reviewing SDG-implementation, involving audit institutions, and including budgeting services (see also [18]).

**Fig 3:**
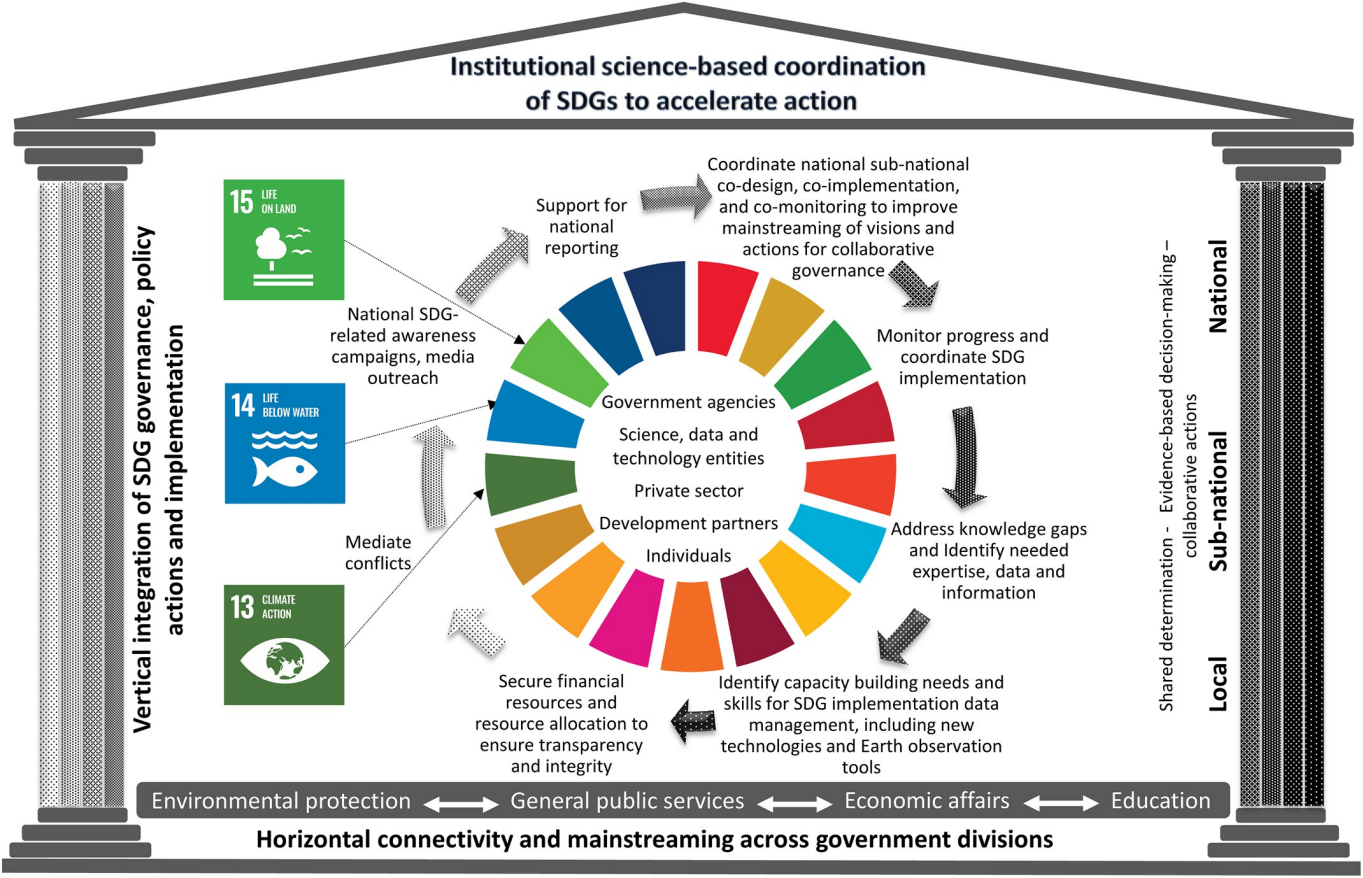
Graphic showing potential outcomes of a national, centralised, independent scientific and technical coordination body for implementing the SDGs and fast-tracking actions. Sources: Fig 3 draws upon recent insights from IPBES, IPCC, and UNCCD on institutional responses to climate change (SDG 13) and unsustainable utilization and degradation of marine (SDG 14) and land resources (SDG 15), as well as experiences from Finland [50] and Taiwan [51]. Eurostat Classification -definitions of the functions of government are used to show how horizontal connectivity and mainstreaming across government divisions can be improved for environmental protection, general public services, economic affairs, and education, categorizing national functions [52] and areas of activity that support a whole-of-government approach, through a national, centralised, independent scientific and technical coordination body.

Drawing together the gaps, needs and existing strengths of current approaches, to coordinate complex vertical national institutional set-ups (which also include *“collaboration between different levels of government*, *and engagement with stakeholders”* [18], especially with regard to optimizing operations to enable fast-tracking SDG-implementation), a national, centralised, independent body with scientific and technical competencies (including digital innovations, geospatial technologies and social sciences expertise) potentially offers many advantages [49] (Fig 3).

**Fig 4:**
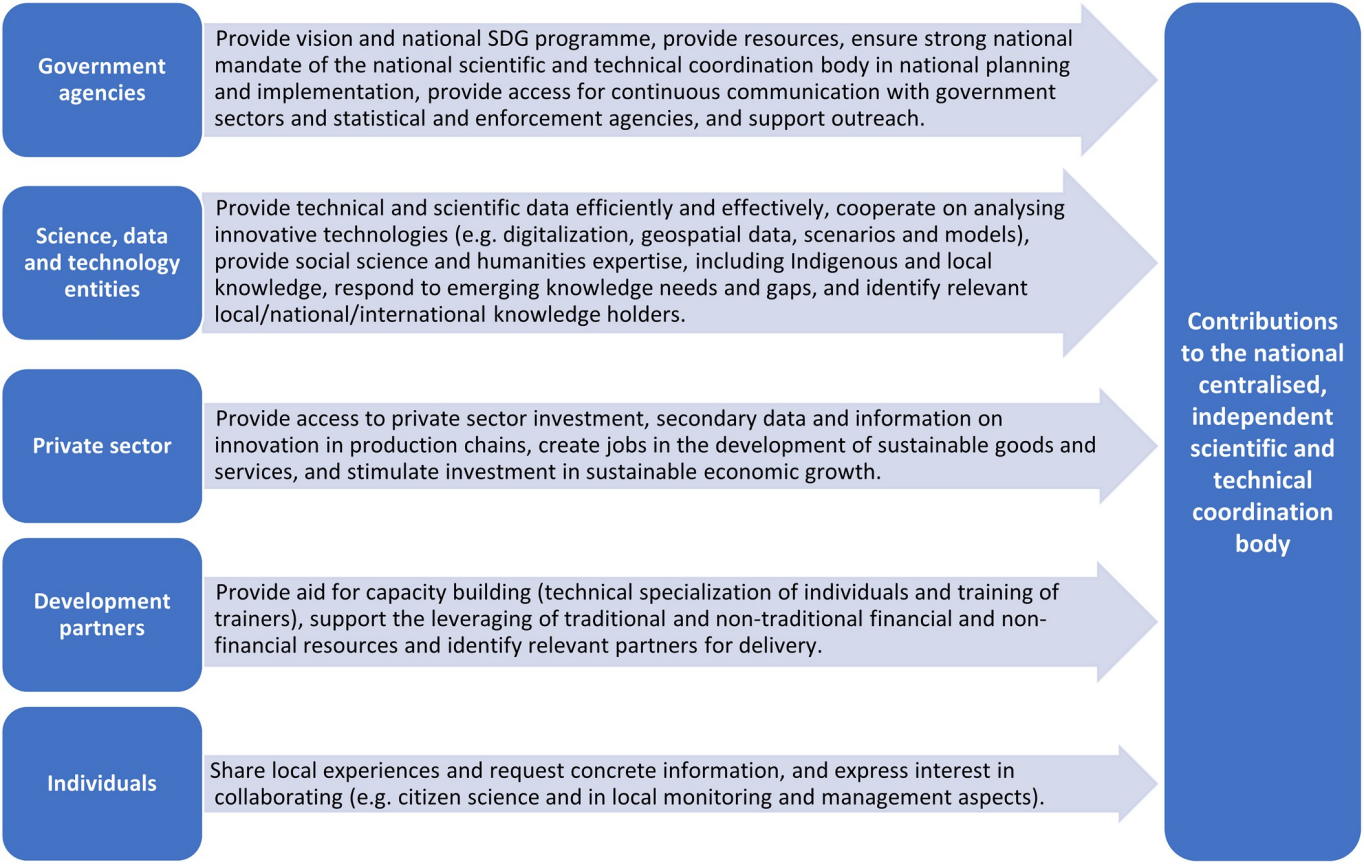
Schematic showing illustrative contributions that different groups could make toa national centralised, independent scientific and technical coordination body. Indicated stakeholder contributions are not indended to be exhaustive, but rather encourage countries to further develop their contributions according to their national circumstances.

The kind of coordination body proposed should have the potential to provide evidence-based data and information by collecting and synthesizing data and monitoring knowledge, but also by analyzing and using proven and new technologies (e.g. means of digitalization or geospatial data). To ensure effective operationalization for evidence-based decision-making, social equity and justice must also be considered, requiring social science as well as local and traditional inputs. Fig 3 further shows that moving towards evidence-based and coherent approaches to accelerate sustainable and integrated development requires a collective collaborative governance approach. This would take into account the requirement to incorporate the diverse needs and values of society, through partnership building with both individuals and groups, which could support national aspirations in the development of a national centralized, independent scientific ad technical coordination body for SDG implementation. (See also [53]) who describes the iterative application of a framework to harness effective collaborations and partnerships to implement the SDGs). Such societal engagement could inspire and energize coordinated national actions that support, but do not depend on policy representation [51, 54–56] helping to create a shared whole-of-society commitment to evidence-based collaborative action to advance shared goals and visions [57] (Fig 4). Pooling expertise ensures that the necessary knowledge for the co-design, co-implementation and co-monitoring of measures is bundled, thereby reducing trade-offs during implementation [51]. It also supports the timely scaling up and scaling out of best practices (see also [58]). In turn, this would help to monitor the performance of government and support public activities, improving accountability and reporting of national achievements in progress on SDG goals and targets.

## Discussion and conclusion

This paper has taken scientific reports developed for various science-policy interfaces to identify deficiencies in national institutional arrangements that restrict SDG implementation. These findings were then linked to in-depth analysis of 93 country reports to connect the science to ongoing national institutionalization processes for SDG implementation. This helped identify how deficiencies can be addressed, as well as how institutional changes can support national level fast-tracking of SDG implementation processes, including the key role of subnational governments, cities and local authorities (also underscored in [59]). Our findings indicate that governments seem most adept with horizontal engagement across ministries, but this tends to encourage prioritization of particular drivers, motivations and values and does not necessarily lead to a whole-of-government approach. This is not surprising (see also [18]). IPBES [60] notes that in spite of the diversity of values associated with nature, policymaking usually prioritizes only a subset (generally those values that focus on economic development or growth), yet underscores that “sustainable and just futures require institutions that enable a recognition and integration of diverse values of nature and nature’s contributions to people” [60]. This shows the vital role that private and financial sectors play among other actors in society, including Indigenous people and local communities, in biodiversity conservation, ecosystem restoration and sustainable use to achieving transformative change [59]. Recognition of the need for engagement with academic, science and technology actors is therefore central for evidence-informed SDG implementation. It is also vital in fast-tracking measures that engage a wider range of stakeholders and their values, both horizontally and vertically, supporting not just a whole-of-government but a whole-of-society approach. While some countries are taking steps towards such engagement, this is not yet widespread and power asymmetries can be problematic. IPBES emphasizes that “… power shapes the extent to which the values held by different actors are considered in decision-making. Institutions that enable more diverse values to be considered have greater potential to avoid or mitigate conflicts, as these often arise from not identifying and anticipating value clashes” [60].

Operationalizing the series of parallel processes identified in the previous section which enable inclusive horizontal and vertical actions therefore indicates that the capacities of a national centralised, independent scientific and technical coordination body for coordinating SDG-implementation should include experience in participatory and stakeholder inclusion processes. Mediation abilities would also be required to negotiate differences or trade-offs that could emerge from different perceptions and interests and to even proactively prevent or mitigate the emergence of such conflicts [61, 62], saving time and other resources required for efficient SDG implementation. Mediation or deliberation abilities may also be required in areas of action where data and information are poor or absent. In these situations, the risk of igniting controversies between demands and interests of different stakeholders could prevent successful fast-tracking because action on the ground may not be backed by sufficient scientific certainty. At the same time, a national centralised, independent scientific and technical coordination body for SDG implementation may also require regular institutional self-assessment of its abilities, to ensure it is fit for purpose and adequately resourced (Table 2).

**Table 2:** Essential competencies to be considered during a self-assessment of a national, centralised, independent scientific and technical coordination body for SDG implementation to ensure fitness for purpose.

Abilities and capacities to:
Attract expertise and competence Mediate and network between different stakeholders Regularly monitor its policies and practices to ensure clarity, accuracy, effectiveness and integrity Change if necessary, procedures and practices Calendar and monitor actions Use existing or developing standards for design, implementation, monitoring and reporting Communicate its needs Report on progress in required and other relevant formats

Source: [47]

In a limited resource and budgetary context but where synergies in actions that support collaborative, inclusive, whole-of-society approaches, alongside horizontal and vertical interlinkages are enabled, governments could usefully draw on academia or other independent (non-governmental) actors to fulfil the role of national, centralised, independent scientific and technical coordination body for SDG implementation. This would capitalise on those institutions that already exist to enhance implementation of the SDGs, support progress beyond 2030 and should actively enable the systematic inclusion of Indigenous and local knowledge [32, 63]. Such an approach would nevertheless require the power asymmetries noted earlier between different institutions to be acknowledged and managed, alongside more widespread appreciation for other, non-scientific forms of knowledge and evidence.

While our findings point toward solutions to enhance national institutions to fast-track measures to progress towards the SDGs, and various fast-tracking measures were bundled into co-design and co-implementation categories (Table 1), short-cuts are not a substitute for coordinated and integrated planning of SDG implementation and their continuation over the long term. Ongoing efforts, alongside acceleration, will be vital to realise action and fast-track progress towards sustainable development. Such efforts may include looking beyond the land and environmental sector to identify further lessons that can be transferred from elsewhere.

## Supporting information

S1 Table Selected quotes from science-policy reports that highlight the need for institutional anchors for stakeholder engagement and participation to support SDG implementation. (DOCX)

S2 Table Specific mention of the collaboration of national arrangements for the implementation of the 2030 agenda with the academia and science in national arrangements. The intensity of collaboration can vary considerably. Only countries marked * specifically mention academia, institutions or government departments with specific scientific and/or technical skills in their national arrangements concerning “political guidance and oversight”; “lead and coordination”, and/or “implementation of SDGs”.
(DOCX)

S3 Table National institutional efforts that give science more responsibilities in domestic SDG processes. (DOCX)

S4 Table Minimum set of criteria used by UNDESA to select SDG Acceleration Actions [1]. (DOCX)
