# Supplementary material for: Fast-tracking action on the Sustainable Development Goals by enhancing national institutional arrangements
Source: PLoS One. 2024 Mar 20;19(3):e0298855. doi: 10.1371/journal.pone.0298855 (PMC10954137; doi:10.1371/journal.pone.0298855)
Supplement: S1 Table — (DOCX) [file pone.0298855.s001.docx]

**Table S1: Selected quotes from science-policy reports that highlight the need for institutional anchors for stakeholder engagement and participation to support SDG implementation**

| **Source** | **Quote** |
| --- | --- |
| [1] | - “Achieving land degradation neutrality depends on the integration of multiple responses across local, regional and national scales and across multiple sectors including agriculture, pasture, forest and water.” (p. 20) - “Addressing desertification, land degradation, and food security in an integrated, coordinated and coherent manner can assist climate resilient development and provides numerous potential co-benefits.” (p. 30) - “Successful management of trade-offs often includes maximising stakeholder input with structured feedback processes, particularly in community-based models, use of innovative fora like facilitated dialogues or spatially explicit mapping, and iterative adaptive management that allows for continuous readjustments in policy as new evidence comes to light” (p. 30). - “The effectiveness of decision-making and governance is enhanced by the involvement of local stakeholders (particularly those most vulnerable to climate change including indigenous peoples and local communities, women, and the poor and marginalised)” (p. 30). - “Integration across sectors and scales increases the chance of maximising co-benefits and minimising trade-offs” (p. 30) - “Sustainable land management in the context of climate change is typically advanced by involving all relevant stakeholders in identifying land-use pressures and impacts … as well as preventing, reducing and restoring degraded land” (p. 31) - “Inclusiveness in the measurement, reporting and verification of the performance of policy instruments can support sustainable land management.” (p. 31) - “Involving stakeholders in the selection of indicators, collection of climate data, land modelling and land-use planning, mediates and facilitates integrated landscape planning and choice of policy” (p. 31) - “Agricultural practices that include indigenous and local knowledge can contribute to overcoming the combined challenges of climate change, food security, biodiversity conservation, and combating desertification and land degradation.” (p. 31) - Coordinated action across a range of actors including businesses, producers, consumers, land managers and policymakers in partnership with indigenous peoples and local communities enable conditions for the adoption of response options.” (p. 31) - “Empowering women can bring synergies and co-benefits to household food security and sustainable land management.” (p. 31) |
| [2] | - “Unless urgent and concerted action is taken, land degradation will worsen in the face of population growth, unprecedented consumption, an increasingly globalized economy and climate change.” (p.13) - “Land managers, including indigenous peoples and local communities, as well as experts and other knowledge holders, all have key roles to play in the design, implementation and evaluation of more sustainable land management practices.” (p. 14) - “Achieving success requires selecting from the full toolkit of approaches that have been effectively implemented in different biophysical, social, economic and political settings. Such a toolkit includes a wide range of low-impact farming, pastoral, forest management and urban design practices based on scientific, indigenous and local knowledge systems.” (p. 15) - “Integrating different practices into landscape-scale planning, including local-level sustainable finance and business practices, can reduce the impacts of degradation and enhance the resilience of both ecosystems and rural livelihoods.” (p. 15) - “Participatory planning and monitoring, based on, among others, land capabilities that include local institutions and land users are supported by multiple knowledge and value systems, are more likely to result in agreement among stakeholders and the effective implementation and monitoring of integrated land management plans.” (p. 15) |
| [3] | - “Nature can be conserved, restored and used sustainably while other global societal goals are simultaneously met through urgent and concerted efforts fostering transformative change.” (p. 16) - “Societal goals, including those related to food, water, energy, health and the achievement of human well-being for all, mitigating and adapting to climate change and conserving and sustainably using nature, can be achieved in sustainable pathways through the rapid and improved deployment of existing policy instruments and new initiatives that more effectively enlist individual and collective action for transformative change.” (p. 16) - “By its very nature, transformative change can expect opposition from those with interests vested in the status quo, but such opposition can be overcome for the broader public good. If obstacles are overcome, a commitment to mutually supportive international goals and targets, supporting actions by indigenous peoples and local communities at the local level, new frameworks for private sector investment and innovation, inclusive and adaptive governance approaches and arrangements, multi-sectoral planning, and strategic policy mixes can help to transform the public and private sectors to achieve sustainability at the local, national and global levels.” (p. 16 f) - “Transformations towards sustainability are more likely when efforts are directed at the following key leverage points, where efforts yield exceptionally large effects: …., (5) justice and inclusion in conservation” (p. 17) - “Risks related to the inevitable uncertainties and complexities in transformations towards sustainability can be reduced through governance approaches that are integrative, inclusive, informed and adaptive.” (p. 17) - “Recognizing the knowledge, innovations, practices, institutions and values of indigenous peoples and local communities, and ensuring their inclusion and participation in environmental governance, often enhances their quality of life and the conservation, restoration and sustainable use of nature, which is relevant to broader society. Governance, including customary institutions and management systems and co-management regimes that involve indigenous peoples and local communities, can be an effective way to safeguard nature and its contributions to people by incorporating locally attuned management systems and indigenous and local knowledge.” (p. 18) |
| [4] | - “To advance a new global land agenda, rights and rewards need to be underpinned by responsibility: increased security of tenure, gender equity, and appropriate incentives and rewards are essential enabling factors to help producers adopt and scale up more responsible land management practices.” (p. 9) - “Smart land use planning is about doing the right thing in the right place at the right scale: a multifunctional landscape approach advocates for more rational land use allocations that lead to greater resource use efficiency and the reduction of waste; it is based on the principles of participation, negotiation, and cooperation.” (p. 9) |

**References**

1. IPCC. Summary for Policymakers. Masson-Delmotte V, Pörtner H-O, Skea J, Calvo Buendia E, Zhai P, Roberts D, et al., editors. Climate Change and Land:and IPCC Special report in climate change, desertification, land degradation, sustainable land management, food security, and greenhouse gas fluxes in terrestrial ecosystems. IPCC; 2019.

2. IPBES. Summary for policymakers. Assessment report on land degradation and restoration of the Intergovernmental Science-Policy Platform on Biodiversity and Ecosystem Services. Bonn, Germany: IPBES Secretariat; 2018.

3. IPBES (Intergovernmental Science-Policy Platform on Biodiversity and Ecosystem Services). Summary for policymakers of the global assessment report on biodiversity and ecosystem services of the Intergovernmental Science-Policy Platform on Biodiversity and Ecosystem Services. [Internet]. Díaz S, Settele J, E.S. .E. S. Brondízo, Ngo HT, Guèze M, Agard J, et al., editors. Intergovernmental Science-Policy Platform on Biodiversity and Ecosystem Services. Bonn, Germany: IPBES Secretariat; 2019. Available from: https://zenodo.org/record/3553579#.YfmYTerMI2w

4. UNCCD. Global Land Outlook 1st Edition. first edit. The Global Land Outlook. Bonn, Germany; 2017.
