# Supplementary material for: Fast-tracking action on the Sustainable Development Goals by enhancing national institutional arrangements
Source: PLoS One. 2024 Mar 20;19(3):e0298855. doi: 10.1371/journal.pone.0298855 (PMC10954137; doi:10.1371/journal.pone.0298855)
Supplement: S2 Table — The intensity of collaboration can vary considerably. Only countries marked * specifically mention academia, institutions or government departments with specific scientific and/or technical skills in their national arrangements concerning “political guidance and oversight”; “lead and coordination”, and/or “implementation of SDGs”. (DOCX) [file pone.0298855.s002.docx]

**Table S2 Specific mention of the collaboration of national arrangements for the implementation of the 2030 Agenda with the academia and science in national arrangements.** The intensity of collaboration can vary considerably. Only countries marked * specifically mention academia, institutions or government departments with specific scientific and/or technical skills in their national arrangements concerning “political guidance and oversight”; “lead and coordination”, and/or “implementation of SDGs”. Information sources: [1,2]

| **Country** | **Source** | **Degree of engagement of academia in national SDG implementation** |
| --- | --- | --- |
| Albania* | [2] | In 2017, 25 universities signed a pledge to advance the 2030 Agenda. The high-level Inter-Ministerial Committee on the SDGs includes representatives of these signatories |
| Algeria* | [1] | National awareness raising campaigns promote engagement with academic and scientific communities. The Ministry of Higher Education and Scientific Research has organized awareness raising workshops and training on the SDGs |
| Armenia* | [2] | Creation of a National SDG Innovation Lab |
| Australia | [2] | The Department of Foreign Affairs and Trade engages with academia and higher education institutions (e.g. the Research for Development Impact Network and the Sustainable Development Solutions Network). Research and expertise are provided by the Commonwealth Scientific and Industrial Research Organization (CSIRO) |
| Azerbaijan | [1] | The National Coordination Council on Sustainable Development has been in contact with academia |
| Bahamas* | [2] | A planned SDG Technical Committee will include non-governmental stakeholders, e.g. academia |
| Bahrain | [2] | The SDGs are implemented through the Government Plan of Action, in partnership with various non-governmental experts, including academia |
| Cabo Verde* | [2] | The implementation mechanism of the national Sustainable Development Strategic Plan 2017-2021 will engage with different non-governmental stakeholders, including academia |
| Colombia* | [2] | A High-Level Inter-institutional Commission (SDG Commission) established in 2015 oversees national adaptation and implementation of the SDGs. It includes the national Science, Technology and Innovation Department, and is responsible for coordinating relevant stakeholders, including academia |
| Congo (Rep. of the) | [1] | National multi-stakeholder meetings to discuss the implementation of the 2030 Agenda included academia |
| Côte d’Ivoire* | [1] | The National Steering Committee on SDGs is led by the Ministry of Planning and Development, including a broad spectrum of entities from e.g. civil society and academia |
| Croatia* | [1] | The Steering Committee on the SDGs, headed by the Ministry of Planning and Development includes non-governmental stakeholders, such as academia |
| Dominican Republic* | [2] | The national High-Level Inter-Institutional Commission for Sustainable Development coordinating implementation of the 2030 Agenda, permits representation from non-governmental entities, including Academia |
| Eswatini | [1] | The national SDG technical working team, which meets regularly to discuss issues regarding indicators and implementation, has representatives of non-governmental entities, including academia |
| Greece | [2] | A Research and Innovation Strategy for Smart Specialisation has been established in every region, focusing on regional aspects for sustainable development |
| Guyana | [1] | A consultative multi-stakeholder process was used to develop the national Green State Development Strategy: Vision 2040, which included non-governmental entities, including academia |
| Hungary* | [2] | The National Council for Sustainable Development, chaired by the Speaker of Parliament, meets quarterly, and is responsible for overseeing SDG implementation since 2015. It includes members from academia. In 2017, A new inter-ministerial coordination mechanism was initiated, including academia |
| Indonesia* | [1] | The SDG National Coordination Team includes a Steering Committee chaired by the President, an Implementation Team, a Technical Working Group, and an Expert Team. Teams also include representatives from academia |
| Iraq* | [1] | The Ministry of Planning leads and coordinates a Monitoring Committee, which also involves representatives from non-governmental entities, including academia |
| Ireland | [2] | A national SDG Stakeholder Forum has been established, chaired by the Department of Communications, Climate Action and Environment and including non-government entities, e.g. academia |
| Israel | [1] | An academic institution organised a conference on the SDGs in 2017 |
| Lithuania* | [2] | The National Commission on Sustainable Development is chaired by the Prime Minister. It is in charge of monitoring and implementation of the National Strategy for Sustainable Development, and providing policy advice. It involves non-governmental entities, incl. academia |
| Mauritius* | [1] | An SDG Steering Committee coordinates the implementation of the 2030 Agenda. The Committee also includes representatives from non-government entities, including academia |
| Mexico* | [2] | In 2017 the National Council for the 2030 Agenda for Sustainable Development was established, chaired by the President. The Office of the President serves as secretariat. The Council coordinates actions for SDG implementation, and includes non-governmental entities, including academia |
| Mongolia* | [1] | In 2019, a Working Group on the SDGs, chaired by the Chief of Cabinet Secretariat, was established by instruction of the Prime Minister. It also includes representatives from non-governmental entities, including academia. Multi-stakeholder consultations on the integration of sustainable development principles also included non-governmental entities. In 2017, a Parliamentary Sub-Committee on the SDGs was established under the Standing Committee on Social Policy, Education, Culture and Science to monitor implementation of the Sustainable Development Vision 2030 and the 2030 Agenda |
| New Zealand | [1] | Annual National SDG Summits were organized in 2018 and 2019 and promote awareness-raising and cross-sectoral discussion of the 2030 Agenda. The creation of stakeholder groups to discuss SDG implementation includes Academia |
| Philippines* | [1] | The Development Budget Coordination Committee – Sub-Committee on the SDGs will aid coordination. The Sub-Committee has four technical working groups which complement the work of existing government coordination mechanisms and enable engagement with non-government entities, including academia |
| Romania | [2] | A Subcommittee for Sustainable Development has been established by the Parliamentary Foreign Policy Committee. It has started consultation a process on revising the National Strategy for Sustainable Development in line with the SDGs. This process also includes non-governmental entities, including academia |
| Saint Lucia* | [1] | The SDG National Coordinating Committee oversees SDG implementation and monitoring. It is co-chaired by ministries considered relevant to sustainable development and meets quarterly. It includes representatives from non-governmental entities, including academia |
| Slovakia* | [2] | The Government Council for the 2030 Agenda for Sustainable Development is chaired by the Deputy Prime Minister for Investments and Informatization and coordinates policy processes related to the SDGs. The Council also includes representatives of non-governmental entities, including academia |
| South Africa* | [1] | The University of Pretoria hosts the South African SDG Hub, an online platform for exchanging SDG resources and experiences |
| Spain | [2] | The creation of a Sustainable Development Council should provide a formal institutional framework for consultations on the 2030 Agenda between a range of non-governmental entities, including academia |
| Sri Lanka | [2] | The Consultative Committee on Sustainable Development includes representatives from non-governmental entities, including academia |
| State of Palestine* | [2] | A National SDG Team involving government and non-government entities coordinates SDG implementation and monitoring. The Team is led by the Prime Minister’s Office, and supported by multi-stakeholder SDG Working Groups |
| Sudan* | [2] | The High-Level National Coordination Mechanism includes a range of stakeholders, including non-government entities such as academia |
| Switzerland | [2] | The national 2030 Agenda Advisory Group addressing policy-making involves representatives of non-governmental entities, including academia |
| Timor Leste* | [1] | The Office of the Prime Minister chairs a Working Group on the SDGs, created in 2015, and includes representatives of non-governmental entities, e.g. academia |
| Tunisia | [1] | A pilot program, initiated by the Governorate of Médenine supports the regional appropriation of the SDGs. Non-governmental entities, including academia, were also involved in development of the respective Five-Year Regional Development Plan |
| Turkey | [1] | A National SDG Best Practices database was established in 2019, including practices from non-governmental entities (e.g. academia). To strengthen understanding and integration of the 2030 Agenda, a training on the SDGs was developed which also targeted a range of non-governmental actors |
| Turkmenistan* | [1] | In 2017, an Interagency Working Group was established to oversee SDG implementation, including deputy heads of ministries and government agencies and non-governmental entities (e.g. academia). The Government also created a Scientific and Methodological Center on SDGs to monitor implementation, hosted in the Institute of International Relations under the Ministry of Foreign Affairs |

**References**

1. UNDESA (United Nations Department of Economic and Social Affairs). Compendium of National Institutional Arrangements for implementing the 2030 Agenda for Sustainable Development: The 47 countries that presented voluntary national reviews at the high political forum in 2019 [Internet]. 2019. Available from: https://sustainabledevelopment.un.org/content/documents/22008UNPAN99132.pdf

2. UNDESA (United Nations Department for Economic and Social Affairs). Compendium of National Institutional Arrangements for implementing the 2030 Agenda for Sustainable Development: The 46 countries that presented voluntary national reviews at the high-level political forum in 2018 [Internet]. 2018. Available from: https://sustainabledevelopment.un.org/content/documents/25839Compendium_of_National_Institutional_Arrangements.pdf
