# Supplementary material for: Fast-tracking action on the Sustainable Development Goals by enhancing national institutional arrangements
Source: PLoS One. 2024 Mar 20;19(3):e0298855. doi: 10.1371/journal.pone.0298855 (PMC10954137; doi:10.1371/journal.pone.0298855)
Supplement: S3 Table — (DOCX) [file pone.0298855.s003.docx]

**Table S3 National institutional efforts that give science more responsibilities in domestic SDG processes**

| **Country** | **Source** | **Acknowledgement of the need for an institutionalized central coordinating system for national SDG implementation** |
| --- | --- | --- |
| Armenia | [2], see also [website](https://www.am.undp.org/content/armenia/en/home/projects/armenia-national-sdg-innovation-lab.html) | Armenia, in cooperation with the United Nations and with support from the Russian Federation and Sweden, set up a National SDG Innovation Lab to utilize data and statistics for “evidence-based policy-making”, and provide insights into human behaviour to assess the effectiveness of policy options for “evidence-based policy-making”. The Lab strives to provide: (1) Public Policy Innovation through research and development recommendations; (2) Capacity building to create a “network of SDG Champions, spread across sectors”; and (3) “non-traditional financing mechanisms to […] shift from funding to financing for development”. Despite these ambitious goals, that the project is planned to last only 32 months, so an evaluation of its value for SDG-implementation over the longer term may be limited. |
| Azerbaijan | [1]: 17f | Azerbaijan for instance has developed an “Innoland incubation and acceleration centre” to support start-ups with technologies and innovative solutions to address SDGs. |
| Colombia | [2]: 32 | Colombia has created a “High-Level Interinstitutional Commission” which is managed by the National Planning Department to oversee the adaptation and implementation of the SDGs, and to promote coordination between different stakeholders, including academia as one stakeholder group. The national Science, Technology and Innovation Department is also engaged in the Commission. |
| Hungary | [2]: 49 | In 2008, the Hungarian Parliament established the multi-stakeholder “National Council for Sustainable Development”, which is chaired by the Speaker of the Parliament, and includes the academia to provide independent advice and to oversee sustainable development issues. Since 2015, it is also responsible for overseeing the SDG implementation. The economic working group is led by the Ministry of Finance, the social working group is led by the Secretariat of Social Action, and the environmental working group is led by the Secretariat of the Environment. |
| Indonesia | [1]: 59 | The Ministry of National Development Planning established the “Indonesian SDGs Academy” to provide capacity building for national and local stakeholders for SDG implementation. |
| South Africa | [1]:112 | The South African SDG Hub is an online platform hosted by the University of Pretoria, through which policymakers, civil society, the private sector, and academia can share resources and experiences regarding the SDGs. |
| Turkmenistan | [1]:127 See also [website](http://tdh.gov.tm/news/en/articles.aspx&article8626&cat30) | The Government of Turkmenistan has set up a Scientific and Methodological Center for the Sustainable Development Goals at the Institute of International Relations of the Ministry of Foreign Affairs, to monitor and ensure implementation of the 2030 Agenda and conduct supportive scientific and methodological work. |

**References**

1. UNDESA (United Nations Department of Economic and Social Affairs). Compendium of National Institutional Arrangements for implementing the 2030 Agenda for Sustainable Development: The 47 countries that presented voluntary national reviews at the high political forum in 2019 [Internet]. 2019. Available from: https://sustainabledevelopment.un.org/content/documents/22008UNPAN99132.pdf

2. UNDESA (United Nations Department for Economic and Social Affairs). Compendium of National Institutional Arrangements for implementing the 2030 Agenda for Sustainable Development: The 46 countries that presented voluntary national reviews at the high-level political forum in 2018 [Internet]. 2018. Available from: https://sustainabledevelopment.un.org/content/documents/25839Compendium_of_National_Institutional_Arrangements.pdf
