# Supplementary material for: Fast-tracking action on the Sustainable Development Goals by enhancing national institutional arrangements
Source: PLoS One. 2024 Mar 20;19(3):e0298855. doi: 10.1371/journal.pone.0298855 (PMC10954137; doi:10.1371/journal.pone.0298855)
Supplement: S4 Table — (DOCX) [file pone.0298855.s004.docx]

**Table S4 Minimum set of criteria used by UNDESA to select SDG Acceleration Actions**

| **#** | **Criteria** |
| --- | --- |
| 1 | Facilitate and accelerate implementation of the 2030 Agenda and the SDGs, reflect interlinkages among goals and contribute to policy coherence |
| 2 | Respect principles of the United Nations Charter and the 2030 Agenda |
| 3 | Build on existing successful efforts/initiatives (scaling up, new phase, etc.) or introduce new ones |
| 4 | Include reasonable means of implementation such as finance, technology or capacity building as an element to help ensure longevity and sustainability of the initiative |
| 5 | Commitment or Initiative defined is “evaluable”, based on an adequate SMART (specific, measurable, achievable resource based, and time-based) set of objectives with specified performance indicators, baselines, targets and data sources as needed |
| 6 | Provide access to additional information on the actions (e.g. website, contacts) |

Source: [1]

**References**

1. UN Department of Economic and Social Affairs. About the SDG accelerations. https://sdgs.un.org/partnerships/action-networks/acceleration-actions/about.
